# Assessment of the Association of COPD and Asthma with In-Hospital Mortality in Patients with COVID-19. A Systematic Review, Meta-Analysis, and Meta-Regression Analysis

**DOI:** 10.3390/jcm10102087

**Published:** 2021-05-13

**Authors:** Felix M. Reyes, Manuel Hache-Marliere, Dimitris Karamanis, Cesar G. Berto, Rodolfo Estrada, Matthew Langston, George Ntaios, Perminder Gulani, Chirag D. Shah, Leonidas Palaiodimos

**Affiliations:** 1 Division of Pulmonary Medicine, Montefiore Medical Center, Bronx, NY 10461, USA; freyesv@montefiore.org (F.M.R.); cshah@montefiore.org (C.D.S.); 2 Albert Einstein College of Medicine, Bronx, NY 10461, USA; bertomc@nychhc.org (C.G.B.); matthew.langston@nychhc.org (M.L.); perminder.gulani@nychhc.org (P.G.); leonidas.palaiodimos@nychhc.org (L.P.); 3 Department of Medicine, Jacobi Medical Center, Bronx, NY 10461, USA; 4 Department of Economics, University of Piraeus, 18534 Attica, Greece; dkaramanis@hotmail.com; 5 Division of Pulmonary Diseases and Critical Care Medicine, University of Texas Health at San Antonio, San Antonio, TX 78229, USA; rodolfo_estrada1@yahoo.com; 6 Department of Internal Medicine, Faculty of Medicine, School of Health Sciences, University of Thessaly, 38221 Larissa, Greece; gntaios@med.uth.gr; 7 Division of Hospital Medicine, Jacobi Medical Center, Bronx, NY 10461, USA

**Keywords:** chronic obstructive pulmonary disease (COPD), asthma, COVID-19, meta-analysis, mortality

## Abstract

Together, chronic obstructive pulmonary disease (COPD) and asthma account for the most common non-infectious respiratory pathologies. Conflicting preliminary studies have shown varied effect for COPD and asthma as prognostic factors for mortality in coronavirus disease 2019 (COVID-19). The aim of this study was to explore the association of COPD and asthma with in-hospital mortality in patients with COVID-19 by systematically reviewing and synthesizing with a meta-analysis the available observational studies. MEDLINE, Scopus, and medRxiv databases were reviewed. A random-effects model meta-analysis was used, and I-square was utilized to assess for heterogeneity. In-hospital mortality was defined as the primary endpoint. Sensitivity and meta-regression analyses were performed. Thirty studies with 21,309 patients were included in this meta-analysis (1465 with COPD and 633 with asthma). Hospitalized COVID-19 patients with COPD had higher risk of death compared to those without COPD (OR: 2.29; 95% CI: 1.79–2.93; I^2^ 59.6%). No significant difference in in-hospital mortality was seen in patients with and without asthma (OR: 0.87; 95% CI: 0.68–1.10; I^2^ 0.0%). The likelihood of death was significantly higher in patients with COPD that were hospitalized with COVID-19 compared to patients without COPD. Further studies are needed to assess whether this association is independent or not. No significant difference was demonstrated in COVID-19-related mortality between patients with and without asthma.

## 1. Introduction

Together, chronic obstructive pulmonary disease (COPD) and asthma account for the most common non-infectious respiratory pathologies [1,2,3]. COPD represents the fourth cause of death in the world and is characterized by an irreversible airway obstruction caused by a mix of airway inflammation and parenchymal destruction. This happens commonly after exposure to particles or gases, most commonly associated with smoking [4]. The World Health Organization (WHO) estimates a worldwide prevalence of COPD at about 265 million people, and the general prevalence of moderate to severe COPD in 65 million people [1]. Asthma is characterized by chronic airway inflammation that causes reversible obstruction of airflow [5]. The WHO estimates that approximately 339 million people live with asthma worldwide, and that almost 500,000 yearly deaths can be attributed to asthma [1]. 

Respiratory viral infections are an important cause of asthma and COPD exacerbations [6,7,8]. Coronaviruses are frequently listed as pathogens found in the airway of COPD patients during exacerbations [9,10,11]. In a systematic review involving 1728 patients, coronaviruses were found in 4% of patients with COPD exacerbations [12]. Likewise, coronavirus infections seem to be involved in 2 to 8% of acute asthma exacerbations [13,14,15,16,17]. 

Coronavirus Disease 2019 (COVID-19), a disease caused by infection with severe acute respiratory syndrome coronavirus 2 (SARS-CoV-2), reached the level of pandemic rapidly and caused almost 3 million deaths worldwide within a year (WHO) [18]. Given the facts that COPD and asthma have high prevalence and morbidity and COVID-19 has mainly manifestations from the respiratory system, we hypothesized that patients with COPD and Asthma are at increased risk of death when hospitalized due to COVID-19. Thus, we aimed to systematically review and synthesize with a meta-analysis of available studies the mortality effect of asthma and COPD among hospitalized patients with COVID-19.

## 2. Materials and Methods

This systematic review and meta-analysis was performed following Preferred Reporting Items for Systematic reviews and Meta-analysis (PRISMA) guidelines (Appendix A Table A1) [19].

### 2.1. Literature Search

We conducted a systematic literature search of Medline (U.S. National Library of Medicine, Bethesda, MD, USA), Scopus (Elsevier, Amsterdam, Netherlands) and medRxiv databases (Cold Spring Harbor Laboratory, Suffolk, NY, USA) up to 12 June 2020. We looked for observational studies providing any association between COPD and/or asthma and mortality in hospitalized patients with COVID-19. Two investigators (FRV and MH) independently searched for eligible studies in all databases with manual review of all data points. In cases where there was a disagreement regarding the eligibility of a study, a third investigator (LP) was involved for consensus to be reached.

The search algorithm used to perform our search in Medline was: “COVID-19 AND (mortality OR death OR outcome OR risk OR prognostic OR prognosis) AND (retrospective OR prospective OR cross-sectional OR observational OR original).” Similar algorithms were used in the other two databases. 

The inclusion criteria were: (i) studies with adult patients hospitalized for COVID-19 and (ii) studies that provided association between COPD and/or asthma and mortality by means of frequencies, odds ratio (OR), or relative risk (RR). There was no language restriction in terms of paper selection. Exclusion criteria were: (i) duplicated or overlapping patient populations and (ii) studies that with less than forty patients. For duplicate or overlapping populations, studies with the larger sample size were included.

### 2.2. Data Extraction and Outcomes

Two independent investigators (F.M.R. and M.H.-M.), who were blinded to each other, extracted and recorded in pre-defined sheet form all relevant information from the eligible studies. The following data were extracted: author, study area and design, hospital location, follow-up period, SARS-CoV-2 diagnostic method, sample size, age, sex, race/ethnicity, baseline comorbidities: coronary artery disease (CAD), heart failure, chronic kidney disease (CKD), dyslipidemia, cerebrovascular accident (CVA) or stroke, diabetes, obesity, chronic obstructive pulmonary disease (COPD), asthma, smoking history, and malignancy history. The primary outcome was in-hospital mortality. The secondary outcomes were need for intubation; admission to an intensive care unit (ICU); and mortality of patients admitted to ICU. 

### 2.3. Risk of Bias Assessment

Risk of bias was assessed by two independent reviewers (F.M.R. and M.H.-M.) using the Quality in Prognosis Studies (QUIPS) tool [20]. Studies were assessed as having low, moderate, serious, or critical risk of bias for the following domains: study participation, study attrition, prognostic factor measurement, confounding measurement and account, outcome measurement, analysis, and reporting.

### 2.4. Statistical Analysis

We estimated the odds ratios (ORs) and their respective 95% confidence intervals (CI) for all the individual studies. A meta-analysis using the random effects model was performed using the DerSimonian and Laird method [21]. I-squared test was used to assess for heterogeneity for each outcome among trials. Values < 25% indicated low, 25% to 70% moderate, and >70% high heterogeneity [21]. Publication bias was reviewed with The Egger test and funnel plots. Sensitivity analyses were performed based on the location of the studies. Sensitivity analysis was also performed for the studies that included only general patient population and the studies that provided adjusted effect size estimates. Meta-regression analyses were performed for important covariates and the outcome of mortality to address high heterogeneity among included studies [21]. We defined a statistical significance level of 5% with 95% Confidence Interval. All Statistical analysis was performed with Stata 14.1 (Stata Corp., College Station, TX, USA).

## 3. Results

### 3.1. Search Results

**Figure 1:**
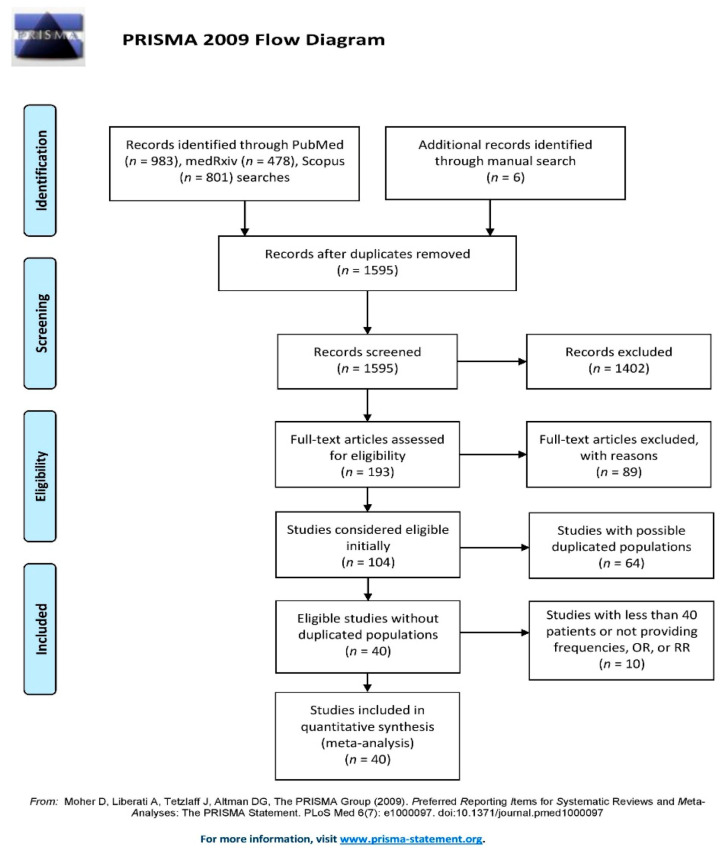
PRISMA Flow Diagram.

**Figure 2:**
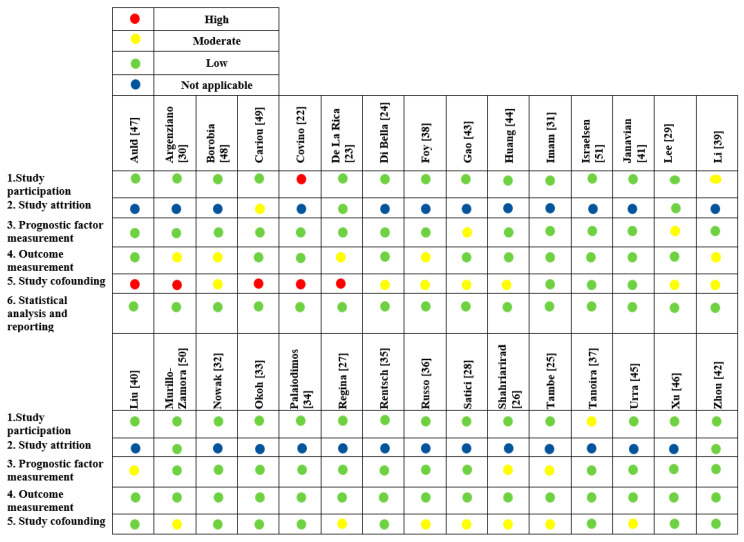
Quality in Prognosis Studies—QUIPS Tool: Risk of Bias Assessment.

A total of 1595 studies were screened; only thirty observational studies met the final inclusion criteria in the analysis (Figure 1; Table A1) [22,23,24,25,26,27,28,29,30,31,32,33,34,35,36,37,38,39,40,41,42,43,44,45,46,47,48,49,50,51]. Main study characteristics of COPD and asthma analysis cohorts are abridged in Table 1. Globally, low risk of bias was found (Figure 2). Nine studies were conducted in Asia, seven in the United States, one in Latin America and thirteen in Europe. 

### 3.2. Patient Characteristics and Primary Events

A total of 21,309 patients were included; 1465 with COPD and 633 with asthma (Table 2). The mean and/or median age was above 60 years in seventeen studies. In only six studies women accounted for more than 50% of the population. Studies reporting results in event rates found, overall: frequency of death events in patient with COPD was 34.6% (312/903) compared to 18.4% (2991/16,240) in the non-COPD group; overall frequency of death events in patients with history of asthma was 20.7% (68/329) compared to 27.2% (2253/8293) in non-asthma group (four out of 23 studies for COPD and two out of seven for asthma studies did not provide the number of death events in the groups of interest; instead, effect size estimates were provided).

### 3.3. COPD vs. Non-COPD

**Figure 3:**
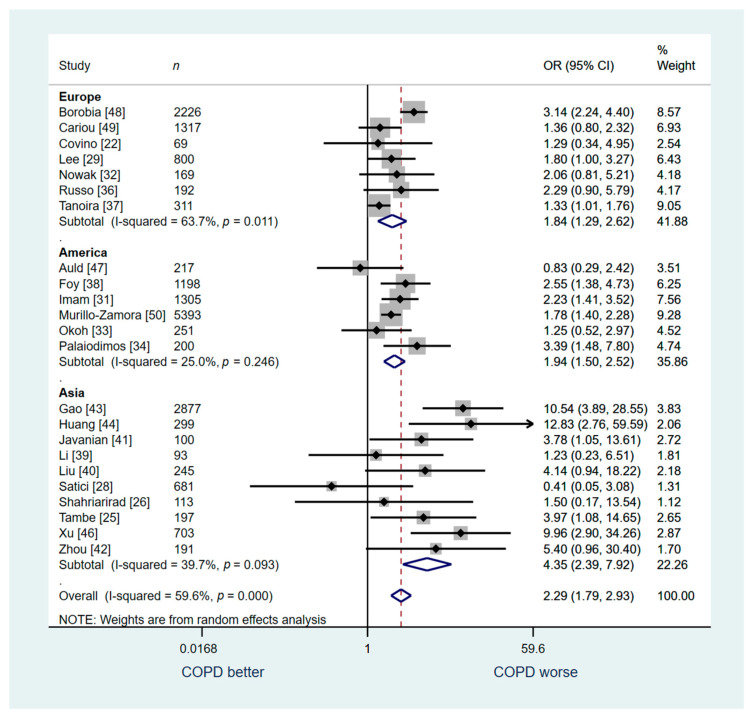
Overall analysis: COPD vs. no COPD for in-hospital mortality.

23 studies (*n* = 19,147) were included in the analysis for the outcome of in-hospital mortality. We found that hospitalized COVID-19 patients with COPD had higher risk of death compared to those without COPD but with moderate overall heterogeneity (OR: 2.29; 95% CI: 1.79–2.93; I2 59.6%; Figure 3). 

A sensitivity analysis was conducted for studies from Europe (*n =* 7), America (*n* = 6), and Asia (*n* = 10). Similar to the overall analysis, our sensitivity analysis for studies conducted in Europe revealed a higher risk of death among patients with COPD compared to those without COPD (OR: 1.84; 95% CI: 1.29–2.62; I^2^ 63.7%, Figure 3). We found similar association for the studies from in America with low heterogeneity (OR: 1.94; 95% CI: 1.50–2.52; I^2^ 25.0%, Figure 3) and among studies conducted in Asia (OR: 4.35; 95% CI: 2.39–7.62; I^2^ 39.7%, Figure 3). 

**Figure 4:**
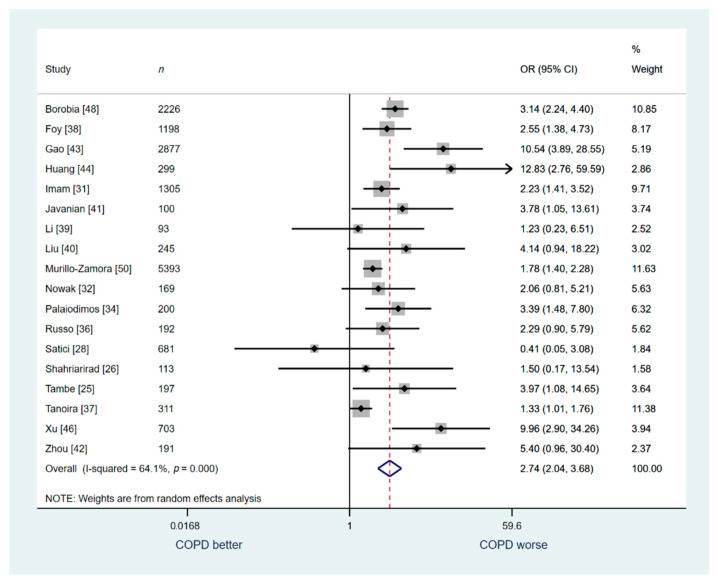
Analysis of the studies that included only general patient population: COPD vs. no COPD for in-hospital mortality.

Another sensitivity analysis was conducted for studies that included general patient population (Studies: 18, *n* = 16,493). Five studies that included a pre-specified special population (e.g., only critically ill patients, only diabetics, only patients with cancer, only a specific age or racial group) were excluded for this analysis. This analysis demonstrated a similar association between COPD and increased risk for in-hospital death (OR: 2.74; 95% CI: 2.04–3.68; I^2^ 64.1%, Figure 4).

Only one study that reported results on the secondary outcome of ICU mortality was included, therefore meta-analysis was not relevant. 

**Figure 5:**
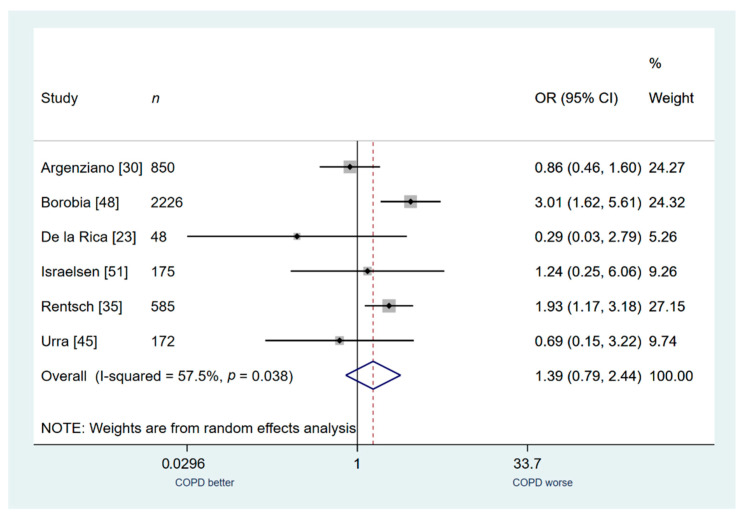
COPD vs. no COPD for the outcome of admission in the ICU.

Six studies (*n* = 4056) were included for secondary outcome analysis of admission to an ICU. COPD patients were not associated with a higher risk of admission to ICU compared to patients without COPD, but a trend was noted (OR: 1.39; 95% CI: 0.79–2.44; I^2^ 57.5%; Figure 5).

**Figure 6:**
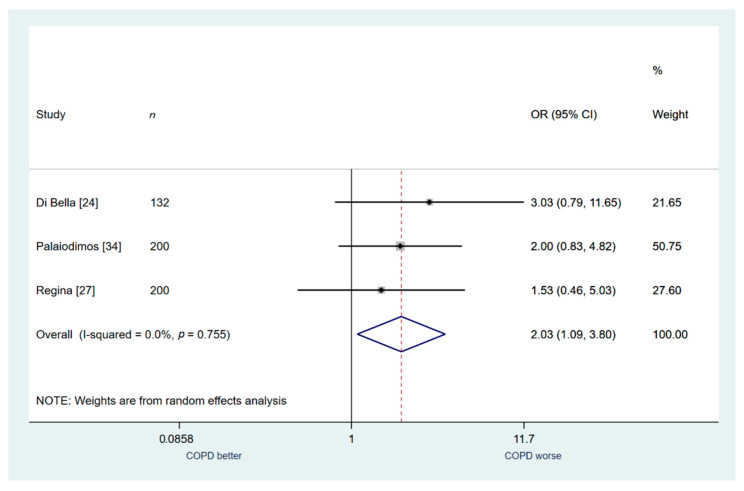
COPD vs. no COPD for the outcome of intubation.

Finally, only three studies (*n* = 532) were included in secondary outcome analysis regarding Intubation. Patients with COPD were found to be associated with a higher risk of intubation compared to patients without COPD (OR: 2.03; 95% CI: 1.09–3.80; I^2^ 0.00%; Figure 6). 

### 3.4. Asthma vs. Non-Asthma

**Figure 7:**
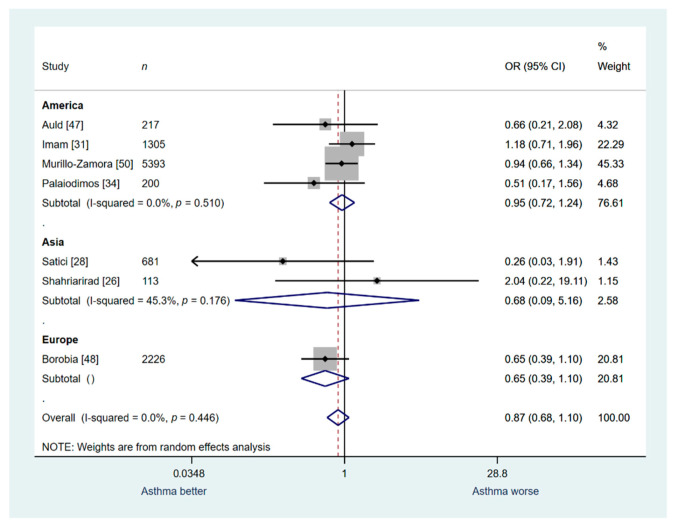
Overall analysis: asthma vs. no asthma for in-hospital mortality.

Seven studies (*n* = 10,136) were included in the secondary outcomes analysis for In-hospital mortality. Asthma patients were not associated with a higher risk of death compared to patients without asthma; the heterogeneity was low (OR: 0.87; 95% CI: 0.68–1.10; I^2^ 0.0%; Figure 7).

A sensitivity analysis was conducted for studies that were performed in Europe (*n* = 1), America (*n* = 4), and Asia (*n* = 2). Similarly, to the overall analysis, the sensitivity analysis based on the continent that the study was conducted revealed no significant association between asthma and in-hospital mortality (Figure 7). 

**Figure 8:**
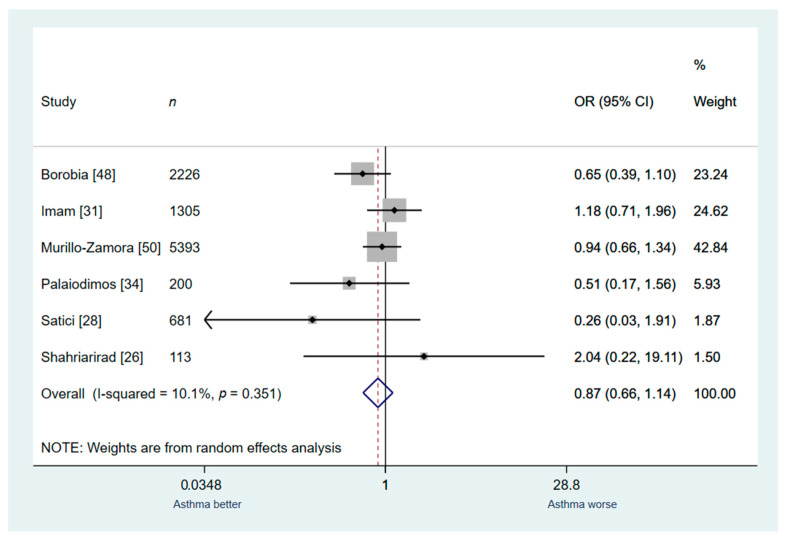
Analysis of the studies that included only general patient population: asthma vs. no asthma for in-hospital mortality.

Another sensitivity analysis was conducted for studies that included general patient population (Studies: 6, *n* = 9918). One study that included a pre-specified special population (only critically ill patients) was excluded for this analysis. Similarly, this analysis demonstrated no association between asthma and risk for in-hospital death (OR: 0.87; 95% CI: 0.66–1.14; I^2^ 0.0%, Figure 8). 

### 3.5. Quality Assessment and Heterogeneity

**Figure A1:**
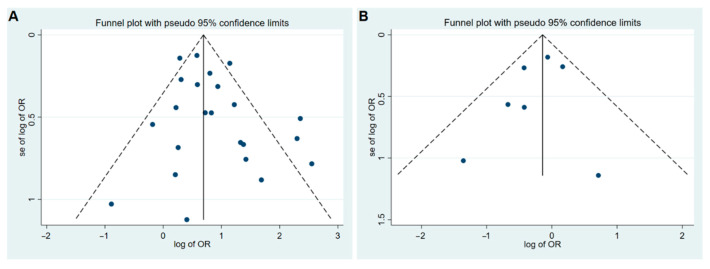
Funnel plots for (**A**) studies included in the COPD analyses; Egger’s test *p* = 0.139 and (**B**) studies included in the asthma analyses; Egger’s test *p* = 0.388.

Eggers’s test and funnel plots were used to assess for publication bias. Visual assessment of the funnel plot was not suggestive for publication bias (Figure A1). Egger test for potential publication bias in a meta-analysis via funnel plot asymmetry was not indicative of publication bias (*p* = 0.388). 

Meta-regression analysis was performed based on several major covariates (age: *p* = 0.838, female sex: *p* = 0.330, hypertension: *p* = 0.743, coronary artery disease: *p* = 0.387, heart failure: *p* = 0.568, chronic kidney disease: *p* = 0.979, diabetes: *p* = 0.924 and malignancy history: *p* = 0.688). No significant interactions were found between the covariates and the outcome of mortality. The results of the meta-regression are found in Table 3.

## 4. Discussion

Our study was a systematic review and meta-analysis of observational studies exploring the association between COPD and asthma with mortality in adult hospitalized patients with COVID-19. The findings of our study are: (i) overall, death was more likely to occur in inpatients with COPD compared with patients without COPD (moderate heterogeneity); (ii) no significant association between history of asthma and in-hospital mortality was revealed.

### 4.1. COPD and Mortality in Patients with COVID-19

Our findings are consistent with the results of an early review that included a total of 2473 patients from studies conducted only in Asia published until late March 2020 [52]. COPD patients were found to have an 88% higher risk of severe disease and 60% higher mortality compared to patients without COPD [52]. Conversely, other larger observational studies have revealed no association of COPD with worse outcomes. Petrilli et al. reported no association between history of COPD and development of critical illness in a cohort of more than 5729 patients from New York [53]. Similarly, a recently published retrospective study of 6916 from California demonstrated no association between history of COPD and in-hospital mortality [54]. Given the heterogeneity of the results across the literature, our meta-analysis attempted to answer this significant question by utilizing a total sample of 19,147 patients across continents.

Seasonal influenza and bacterial pneumonia have been clearly linked to worse outcomes and increased mortality in patients with COPD [55,56,57,58]. On the other hand, the reports regarding the impact of COPD in patients with H1N1 Influenza were contradictory [59,60,61,62,63,64]. Although SARS-CoV and SARS-CoV-2 share 79.6% of their genome, COPD as a comorbidity was underreported during the SARS epidemic of 2003 caused by SARS-CoV, making the comparison difficult [42,65,66,67,68,69,70,71,72,73,74]. 

The higher mortality noted in patients with COPD in our study could be related to smoking, which is considered to be a potential up-regulator of the Angiotensin Converting Enzyme-2 (ACE2) receptor, the adhesion site for the SARS-CoV-2 virus. This hypothesis has been contested [75,76,77,78,79]. COPD has genotypic and phenotypic findings that makes it the prime disease for SARS-CoV-2 targeting. In 2015, Kim et al. showed genes associated with ACE2 level expression were more likely to be found in COPD patients [80], and this has been confirmed by other research groups even in non-smoking COPD patients [76,81,82]. The pathophysiology is presumed to be linked to inflammatory signaling secondary to smoking and to ACE2 expression upregulation in secretory cells expressing ACE2 [82,83,84]. Another possible explanation into why we found patients with COPD to have higher mortality in COVID19 is because they are general sicker than the general population. More so, patients with COPD have more comorbidities than the general population or patients with other chronic diseases [85]; with two to five times higher risk of ischemic heart disease, cardiac dysrhythmia, heart failure, diseases of the pulmonary circulation, and diseases of the arteries [86].

### 4.2. Asthma and Mortality in Patients with COVID-19

Patient with asthma facing an upper respiratory illness would be expected to have a higher risk for a worse outcome when compared to healthy individuals [87]. In the present analysis patients with a history of asthma were not found to have increased in-hospital mortality. Several recent large cohort studies of asthma patients confirm our findings [88,89,90,91]. 

Adults with asthma are at increased risk of invasive pneumococcal infections [92,93], Bacterial pneumonias have been associated with asthma exacerbations but not necessarily with increased mortality [94]. Correspondingly, viral respiratory infections have been linked with acute asthma exacerbations [95,96]. Seasonal influenza increased the risk of acute asthma exacerbation in elderly patients, but, similarly to our findings, no reports have clearly showed increased mortality [97,98,99,100]. Likewise, during the 2009 H1N1 pandemic adult asthmatics were not found to be at higher risk of worse outcomes [4,55,59,101,102,103]. 

Asthma has been shown to have decreased ACE2 expression in murine models [104]. Different asthma phenotypes could have different interactions with the COVID-19 virus: patients with low peripheral blood eosinophils have increased ACE2 expression [105], while ACE2 expression is downregulated in patients with chronic history of respiratory allergies by interleukin modulation [106,107]. This decreased expression of ACE2 throughout the body could explain why asthmatic patients had in general better outcomes than patients with COPD.

### 4.3. Strengths and Limitations

The main strengths of our study are its strict methodology, robust analysis, and large number of included studies and overall patient sample size. Three continents and most of the countries that had high COVID-19 incidence at the time of the analysis were represented. Sensitivity and meta-regression analyses were performed. The main limitations of our study are the observational nature of the primary studies and the lack of spirometry data confirming the diagnosis of obstructive lung disease. COPD is often underdiagnosed in the general population [108]. Another important limitation is the lack of data on the severity of COPD and asthma; thus, we could not estimate the associations of well-controlled versus poorly controlled obstructive lung disease with in-hospital mortality. A patient-level meta-analysis would be needed to assess this particularly important parameter. Moreover, our meta-analysis was limited by moderate heterogeneity, which we tried to rectify by using a random-effects model and performing sensitivity and meta-regression analyses. Important to note, different experimental treatment regimens of COVID-19 across continents may have affected general overall mortality as well as health-care access and level of care to these treatments. Finally, several observational studies have been published after we completed this meta-analysis. However, our study included a large number of studies and patients from different parts of the world, and it is unlikely that the inclusion of the additional recent studies would change our results. 

## 5. Conclusions

In conclusion, this systematic review and meta-analysis showed that patients with history of COPD who were hospitalized with COVID-19 were associated with higher in-hospital mortality. Further prospective studies are needed to assess whether this association is independent or not. In contrast, asthmatic patients with COVID-19 were not found to be associated with higher mortality.

## Figures and Tables

**Table 1:** Characteristics of the included studies. COPD and Asthma analysis cohorts.

Study	Location	Design	Enrollment Start ^#^	Enrollment End ^#^	COPD	Asthma	Sample Size
**Auld** **[47]**	USA	Retrospective	6 March	17 April	Y	Y	217
**Borobia** **[48]**	Spain	Retrospective	25 February	5 April	Y	Y	2226
**Cariou** **[49]**	France	Retrospective	10 March	31 March	Y	N	1317
**Covino** **[22]**	Italy	Retrospective	1 March	31 March	Y	N	69
**Foy** **[38]**	USA	Retrospective	4 March	28 April	Y	N	1198
**Gao** **[43]**	China	Retrospective	1 March	13 March	Y	N	2877
**Huang** **[44]**	China	Retrospective	25 January	24 March	Y	N	299
**Imam** **[31]**	USA	Retrospective	1 March	17 April	Y	Y	1305
**Javanian** **[41]**	Iran	Retrospective	25 February	12 March	Y	N	100
**Lee** **[29]**	UK	Prospective	18 March	26 April	Y	N	800
**Li** **[39]**	China	Retrospective	10 January	22 February	Y	N	93
**Liu** **[40]**	China	Retrospective	1 January	29 February	Y	N	245
**Murillo-Zamora** **[50]**	Mexico	Retrospective	4 March	5 May	Y	Y	5393
**Nowak** **[32]**	Poland	Retrospective	16 March	7 April	Y	N	169
**Okoh** **[33]**	USA	Retrospective	10 March	10 April	Y	N	251
**Palaiodimos** **[34]**	USA	Retrospective	9 March	22 March	Y	Y	200
**Russo** **[36]**	Italy	Prospective	February	April	Y	N	192
**Satici** **[28]**	Turkey	Retrospective	2 April	1 May	Y	Y	681
**Shahriarirad** **[26]**	Iran	Retrospective	20 February	20 March	Y	Y	113
**Tambe** **[25]**	India	Cross-sectional	31 March	24 April	Y	N	197
**Tanoira** **[37]**	Spain	Retrospective	3 March	16 March	Y	N	311
**Xu** **[46]**	China	Retrospective	10 January	13 March	Y	N	703
**Zhou** **[42]**	China	Retrospective	19 December	31 January	Y	N	191
Argenziano * **[30]**	USA	Retrospective	1 March	5 April	Y	Y	850
**De la Rica** **[23]**	Spain	Retrospective	15 March	31 March	Y	N	48
**Israelsen** **[51]**	Denmark	Retrospective	10 March	23 April	Y	Y	175
**Rentsch** **[35]**	USA	Retrospective	8 February	30 March	Y	N	585
**Urra** **[45]**	Spain	Retrospective	1 March	15 April	Y	N	172
**Di Bella** **[24]**	Italy	Prospective	25 March	7 April	Y	N	132
**Regina** **[27]**	Switzerland	Retrospective	1 March	25 March	Y	N	200

Notes: * only the subset of hospitalized patients was included for analysis. ^#^ Year 2020. Abbreviations: USA = United States of America, UK = United Kingdom, COPD = chronic obstructive pulmonary disease, Y = Yes, N = No.

**Table 2:** Baseline characteristics of patients per included studies. Described in absolute and relative frequencies.

Study	Age	Female *n* (%)	Diabetes *n* (%)	Hypertension *n* (%)	CAD *n* (%)	Heart Failure *n* (%)	CKD *n* (%)	CVA *n* (%)	Smoking *n* (%)	Malignancy *n* (%)	COPD *n* (%)	Asthma *n* (%)
**Auld** **[47]**	64 (54–73) ^b^	98 (45.2)	99 (45.4)	134 (61.7)	31 (14.3)	41 (18.9)	58 (26.7)	NA	NA	NA	21 (9.7)	19 (8.8)
**Borobia** **[48]**	61 (46–78) ^b^	1152 (51.8)	381 (17.1)	920 (41.3)	NA	NA	174 (7.8)	NA	157 (7.1)	133 (6.0)	153 (6.9)	115 (5.2)
**Cariou** **[49]**	69.8 (13) ^a^	462 (35.1)	NA	1003 (76.1)	336 (25.5)	140 (10.6)	60 (4.5)	163 (12.4)	447 (33.9)	NA	133 (10.1)	NA
**Covino** **[22]**	84 (82–89 ) ^d^	32 (46.4)	9 (13.0)	41 (59.4)	21 (30.4)	21 (30.4)	NA	20 (29.0%)	NA	3 (4.3)	7 (10.1)	NA
**Foy** **[38]**	NA	534 (44.8)	NA	NA	NA	NA	NA	NA	NA	NA	273 (22.8)	NA
**Gao** **[43]**	NA	NA	387 (13.6)	850 (29.5)	295 (10.25)	23 (0.8)	29 (1.0)	52 (1.8)	190 (6.6)	49 (1.7)	31 (1.1)	NA
**Huang** **[44]**	53.4 (16.7) ^a^	139 (46.5)	35 (11.7)	74 (24.7)	18 (6)	NA	NA	13 (4.3)	48 (16.1)	9 (3.0)	8 (2.6)	NA
**Imam** **[31]**	61.0 (16.3) ^a^	603 (46.2)	393 (30.1)	734 (56.2)	208 (15.9)	75 (5.7)	228 (17.5)	95 (7.3)	356 (27.3)	83 (6.4)	107 (8.2)	115 (8.8)
**Javanian** **[41]**	60.12 (13.87) ^b^	49 (49)	34 (34.0)	31 (31.0)	22 (22.0)	NA	11 (11.0)	3 (3.0)	NA	4 (4.0)	12 (12.0)	NA
**Lee** **[29]**	69 (59.0–76.0) ^b^	349 (43.6)	131 (16.4)	247 (30.9)	NA	NA	NA	NA	NA	800 (100)	61 (7.6)	NA
**Li** **[39]**	51 (17.5) ^a^	52 (55.9)	11 (11.8)	5 (5.4)	4 (4.3)	NA	NA	NA	NA	4 (4.3)	8 (8.6)	NA
**Liu** **[40]**	53.95 (16.9) ^a^	131 (53.5)	23 (9.4)	52 (21.2)	18 (7.3)	NA	NA	NA	10 (4.01)	9 (3.7%)	8 (3.3)	NA
**Murillo-Zamora** **[50]**	NA	1,961 (36.4)	1,677 (31.1)	1,973 (36.6)	NA	NA	299 (5.5)	NA	NA	NA	273 (5.1)	146 (2.7)
**Nowak** **[32]**	63.7 (19.6) ^a^	82 (48.5)	32 (18.9)	80 (47.3)	52 (30.8)	NA	35 (20.7)	58 (34.3)	NA	35 (20.7)	22 (13.3)	NA
**Okoh** **[33]**	62 (49–74) ^b^	122(48.6)	115 (45.8)	175 (69.7)	49 (19.5)	50 (19.9)	46 (18.3)	28 (11.1)	NA	22 (8.7)	23 (9.2)	NA
**Palaiodimos** **[34]**	64 (50.0–73.5) ^b^	102 (51.0)	79 (39.5)	152 (76.0)	33 (16.5)	34 (17.0)	41 (20.5)	22 (11.0)	NA	11 (5.5)	28 (14.0)	27 (13.5)
**Russo** **[36]**	67.7 (15.2) ^a^	77 (40.1)	42 (21.9)	111 (57.8)	26 (13.5)	20 (10.4)	12 (6.3)	16 (8.3)	16 (8..0)	NA	26 (13.5)	NA
**Satici** **[28]**	56.9 (15.7) ^a^	334 (49.0)	191 (28.0)	234 (34.4)	62 (9.1)	19 (2.8)	24 (3.5)	NA	NA	9 (1.3)	28 (4.1)	43 (6.3)
**Shahriarirad** **[26]**	53.75 (16.58) ^a^	42 (37.2)	16 (14.2)	22 (19.5)	16 (14.2)	NA	6 (5.3)	NA	NA	1 (0.9)	9 (7.9)	7 (6.2)
**Tambe** **[25]**	45.8 (17.3) ^a^	107 (54.5)	42 (21.3)	60 (30.5)	4 (2.0)	NA	2 (1.0)	NA	NA	NA	10 (5.1)	NA
**Tanoira** **[37]**	NA	NA	NA	NA	NA	NA	NA	NA	NA	NA	NA	NA
**Xu** **[46]**	46.1 (15.2) ^a^	321(45.6)	64(9.1)	NA	35 (5.0)	NA	10(1.4)	NA	NA	9(1.3)	13 (1.8)	NA
**Zhou** **[42]**	56·0 (46–67) ^b^	72 (37.7)	36 (18.8)	58 (30.4)	15 (7.9)	NA	2 (1.0)	NA	11 (5.8)	2 (1.0)	6 (3.1)	NA
**Argenziano** **[30]**	63.0 (50.0–75.0) ^b^	339 (39.9)	333 (39.2)	525 (61.8%)	115 (13.5)	91 (10.7)	125 (14.7)	72 (8.5)	198 (23.3)	63 (7.4)	56 (6.6)	88 (10.4)
**De la Rica** **[23]**	65.98 (13.9) ^a^	16 (33.3)	11 (22.9)	33 (68.8)	14 (29.2)	NA	8 (16.7)	NA	10 (20.8)	10 (20.8)	5 (10.4)	NA
**Israelsen** **[51]**	71 (55–81) ^b^	90 (51,4)	46 (26.3)	73 (41.7)	90 (51.4)	NA	NA	NA	63 (36.0)	NA	11 (6.3)	20 (11.4)
**Rentsch** **[35]**	66.1 (60.4–71.0) ^b^	27 (4.6)	260 (44.4)	423 (72.3)	163 (27.3)	NA	NA	NA	338 (57.8)	83 (14.2)	90 (15.4)	45 (7.7)
**Urra** **[45]**	NA	68 (39.5)	39 (22.7)	87 (50.6)	28(16.3)	NA	NA	NA	NA	NA	17 (9.9)	NA
**Di Bella** **[24]**	66 (55–75.8) ^b^	42 (31.8)	33 (25.0)	55 (41.7)	24 (18.2)	NA	NA	NA	12 (9.1)	NA	10 (7.6)	NA
**Regina** **[27]**	70 (55–81) ^b^	80 (40.0)	43 (21.5)	87 (43.5)	35 (17.5)	NA	28 (14.0)	21 (10.5)	NA	NA	16 (8.0)	8 (4.0)

Notes: ^a.^ mean ± SD, ^b.^ median (IQR). Abbreviations: CAD = coronary artery disease, CKD = chronic kidney disease, CVA = cerebrovascular accident, COPD = chronic obstructive, SD = standard deviation, IQR = interquartile range, NA = non-available.

**Table 3:** Results of the meta-regression analyses.

	COPD			Asthma		
Variables	Coefficient	Standard Error	*p*-Value	Coefficient	Standard Error	*p*-Value
Age	−0.009	0.006	0.146	0.040	0.175	0.835
Female	0.031	0.013	0.037	−0.026	0.024	0.330
HTN	−0.012	0.008	0.114	−0.004	0.013	0.743
CAD	−0.006	0.014	0.648	0.149	0.148	0.387
HF	−0.016	0.027	0.584	−0.033	0.049	0.568
CKD	−0.015	0.022	0.489	0.001	0.023	0.979
Diabetes	−0.026	0.011	0.023	0.002	0.019	0.924
Malignacy	−0.005	0.005	0.316	0.077	0.173	0.688
CVA	−0.019	0.021	0.371			
Smoking	−0.026	0.013	0.108			

Abbreviations: HTN = hypertension, CAD = coronary artery disease, HF = heart failure, CKD = chronic kidney disease, CVA = cerebrovascular accident. Note: Meta-regression is a meta-analytic tool to examine the impact of moderator variables on study effect size. It cannot be used to evaluate the association of moderator variables with the outcome of interest.

## Data Availability

Data is contained within the article or Supplementary Material. Further patient or study level data supporting reported results are available on request; can be sent as excel sheet file or another statistical file.

## Supplementary Materials

The following are available online at https://www.mdpi.com/article/10.3390/jcm10102087/s1, Table S1: PRSIMA Checklist.

## Appendix A

**Table A1:** Full text articles excluded with reasons.

Exclusion Reasons	*n*
Studies that enrolled special populations (e.g., cancer patients)	12
Studies that did not report separate data on the outcomes of interest	28
Studies that did not specifically report data on COPD and/or asthma	49
